# Conditions on the Mexican moulting grounds influence feather colour and carotenoids in Bullock's orioles (*Icterus bullockii*)

**DOI:** 10.1002/ece3.2836

**Published:** 2017-03-19

**Authors:** Kaitlin L. Sparrow, Kingsley K. Donkor, Nancy J. Flood, Peter P. Marra, Andrew G. Pillar, Matthew W. Reudink

**Affiliations:** ^1^ Department of Biological SciencesThompson Rivers UniversityKamloopsBCCanada; ^2^ Department of ChemistryThompson Rivers UniversityKamloopsBCCanada; ^3^ Migratory Bird CenterSmithsonian Conservation Biology InstituteWashingtonDCUSA

**Keywords:** Bullock's oriole, carotenoid, carryover effects, *Icterus bullockii*, Mexican monsoon region, molt‐migration, plumage, stable isotope

## Abstract

Carotenoid‐based plumage coloration plays a critical role for both inter‐ and intrasexual communication. Habitat and diet during molt can have important consequences for the development of the ornamental signals used in these contexts. When molt occurs away from the breeding grounds (e.g., pre‐alternate molt on the wintering grounds, or stopover molt), discerning the influence of habitat and diet can be particularly important, as these effects may result in important carryover effects that influence territory acquisition or mate choice in subsequent seasons. Several species of songbirds in western North America, including the Bullock's oriole (*Icterus bullockii*), migrate from the breeding grounds to undergo a complete prebasic (post‐breeding) molt at a stopover site in the region affected by the Mexican monsoon climate pattern. This strategy appears to have evolved several times independently in response to the harsh, food‐limited late‐summer conditions in the arid West, which contrast strongly with the high productivity driven by heavy rains that is characteristic of the Mexican monsoon region. Within this region, individuals may be able to optimize plumage coloration by molting in favourable areas characterized by high resource abundance. We used stable isotope analysis (δ^13^C, δ^15^N) to ask whether the diet and molt habitat/location of Bullock's orioles influenced their expression of carotenoid‐based plumage coloration as well as plumage carotenoid content and composition. Bullock's orioles with lower feather δ^15^N values acquired more colorful plumage (orange‐shifted hue) but had feathers with lower total carotenoid concentration, lower zeaxanthin concentration, and marginally lower canthaxanthin and lutein concentration. Examining factors occurring throughout the annual cycle are critical for understanding evolutionary and ecological processes. Here, we demonstrate that conditions experienced during a stopover molt, occurring hundreds to thousands of kilometers from the breeding grounds, influence the production of ornamental plumage coloration, which may carryover to influence inter‐ and intrasexual signaling in subsequent seasons.

## 1 Introduction

Sexual selection is a strong evolutionary force that has been instrumental in shaping the elaboration of ornamental traits and driving genetic divergence and speciation across a range of taxa. Individuals with larger, more elaborate, and more colorful ornamental traits tend to be favoured by sexual selection, as many of these traits act as honest indicators of individual condition or quality and function in inter‐ and intrasexual communication. Plumage coloration, in particular, has garnered considerable attention from evolutionary biologists (Hill & McGraw, 2006) and a preponderance of work has demonstrated the importance of carotenoid coloration for social and sexual signaling.

Carotenoids are bright red, yellow, and orange dietary organic pigments that have a range of functions in animals, including producing color, boosting immune response, and acting as antioxidants (García‐de Blas, Mateo, Guzmán Bernardo, Martín‐Doimeadios, & Alonso‐Alvarez, 2014; Navara & Hill, 2003). To produce feather coloration, carotenoids can be deposited directly from the diet as yellow dietary carotenoids, or they can be modified prior to deposition in feathers. For example, lutein, zeaxanthin, β‐carotene, and β‐cryptoxanthin, the main dietary carotenoids, can be modified to the yellow carotenoids, 3'‐dehydrolutein, canary xanthophyll A&B, or modified to red keto‐carotenoids such as α‐doradexanthin, astaxanthin, canthaxanthin, and adonirubin (McGraw, 2006). The main modifications to dietary carotenoids include oxidation of hydroxyl groups to ketones, which produces modified yellow carotenoids, or the addition of ketones via oxidation, which produces red keto‐carotenoids. These metabolic conversions appear to be condition dependent, indicating that, of all carotenoid‐based coloration, orange/red feathers may be the most honest signals of individual quality and condition (Hill, 2011; Hill & Johnson, 2012; Johnson & Hill, 2013; Hill, 2014; but see Simons, Groothuis, & Verhulst, 2015).

While individual condition is essential for the expression of optimal coloration, individuals may also be constrained by habitat quality during molt. Several studies on great tits (*Parus major*) and Eurasian blue tits (*Cyantistes caeruleus*) have demonstrated the importance of forest composition and quality at a local scale for influencing food/carotenoid availability and thus ultimately plumage coloration (e.g., Slagsvold & Lifjeld, 1985; Arriero & Fargallo, 2006; Eeva, Sillanpää, & Salminen, 2009; but see Ferns & Hinsley, 2008). Broader, regional variation in habitat may also influence regional variation in plumage coloration. For example, house finches (*Haemorhous mexicanus*) exhibit marked geographic variation in the color and extent of carotenoid‐based plumage coloration (Badyaev, Belloni, & Hill, 2012). Diet supplementation experiments revealed that the size of the carotenoid‐based patch resulted from genetic differences between the *frontalis* and *griscomi* subspecies, but that coloration *per se* resulted from differences in carotenoid access during molt. In American redstarts (*Setophaga ruticilla*), males from more northern latitudes express higher red chroma values, which likely reflects higher feather carotenoid content (Norris, Marra, Kyser, Ratcliffe, & Montgomerie, 2007); as for house finches, this geographic variation in plumage coloration was attributed to regional variation in diet and carotenoid availability. These studies demonstrate that molt location and habitat can play an important role in the acquisition of plumage coloration. The effects of habitat may manifest directly, as molting in poor‐quality habitat can result in an insufficient amount of carotenoids being consumed and deposited, and indirectly, through malnourishment, exposure to parasites, high stress load, or other factors. Thus, both direct and/or indirect effects may ultimately result in a need to utilize carotenoids elsewhere or limit the ability of individuals to metabolically convert dietary carotenoids.

Migratory birds vary greatly in the timing and location of their molt. Which feathers are replaced and when they are grown can vary among species and even within species across populations. In the arid West, several species, hereafter referred to as molt‐migrants, interrupt fall migration to molt in the Mexican monsoon region, located in the southwestern United States and northwestern Mexico (Leu & Thompson, 2002; Pillar, Marra, Flood, & Reudink, 2015; Pyle et al., 2009; Rohwer, Butler, Froehlich, Greenberg, & Marra, 2005). This strategy likely evolved due to the extensive green‐up that occurs during the monsoon rains in late summer/early autumn, providing a greater amount of the food resources necessary for molt (Douglas, Maddox, Howard, & Reyes, 1993; Higgins & Gochis, 2007; Rohwer & Manning, 1990; Rohwer et al., 2005). The Mexican monsoon region covers a large geographic area and encompasses a range of habitats, suggesting that individuals may be able to optimize feather coloration by selecting a high‐quality molt location. The challenge, however, is tracking habitat use across seasons in animals moving hundreds or thousands of kilometers between breeding, molting, and wintering areas.

Because habitat can influence food and carotenoid availability, moving to areas with high food availability during molt could provide a strong selective advantage. Although small migratory birds are difficult to track due to their size and the vast distances they travel, stable isotope analysis has become a useful tool for linking different phases of the annual cycle and determining diet and habitat use. Because stable isotopes in feathers are inert once the feathers are grown, analyzing feather isotope values, in particular δ^13^C and δ^15^N, can provide information on diet and habitat use at the time of molt (Wassenaar, 2008; see Methods for details).

Here, we ask whether conditions experienced during molt in the Mexican monsoon region influence feather color and carotenoid content/composition in the molt‐migrant, Bullock's oriole (*Icterus bullockii*). Recently, Pillar et al. (2015) used a combination of geolocators and stable hydrogen isotope analysis to confirm that Bullock's orioles interrupt their southward migration to stopover and molt in the Mexican monsoon region. The authors showed that there was extensive variation in feather hydrogen isotopes, indicating that molt might be occurring across a relatively large geographic area or range of habitats within the Mexican monsoon region. This finding is consistent with low reported molt location fidelity in Bullock's orioles; Pyle et al. (2009) suggest that molt‐migrants, including Bullock's orioles, track resources during the molt period, which implies that individuals may be able to optimize plumage coloration by selecting high‐quality molt locations (i.e., areas where food, and perhaps even carotenoid‐rich food, is abundant). We predicted that Bullock's orioles that molted in high‐quality, wet habitats in the Mexican monsoon region (indicated by low δ^13^C values) would have more carotenoids (especially the red keto‐carotenoid canthaxanthin) deposited in their feathers than those that molted in low‐quality, xeric habitats (high δ^13^C values). However, the relationship between feather carotenoids and δ^15^N could be either positive or negative, depending on the relative richness of dietary carotenoids and the specific biome in which the birds molt.

## 2 Methods

### 2.1 Study species

Bullock's orioles are songbirds that breed as far north as the southern interior of British Columbia, undergo prebasic molt in the Mexican monsoon region, and overwinter from northern Mexico to Central America (Leu & Thompson, 2002; Pillar et al., 2015; Pyle et al., 2009; Rohwer et al., 2005). After second year (ASY) males exhibit bright orange plumage on their breast, belly, rump, and face (the crown, nape, back, scapulars, and lores of males are black, and they possess a bold black eye‐line), while females of all ages have a pale yellow head and breast, with a throat that is black, yellowish, or mixed yellow and black (females with throat patches are hypothesized to be older individuals; Pyle, 1997; Jaramillo & Burke, 1999). Second year (SY) males are similar to females, but also exhibit patches of ASY‐like plumage (Rising, P, & Poole, 1999). In this species, ASY males, who are more orange than SY males, have higher reproductive success (Richardson & Burke, 1999; Williams, 1988), suggesting plumage coloration has an important signaling function.

### 2.2 Fieldwork

Fieldwork was conducted in Kamloops, British Columbia, Canada (50.68 N 120.34 W). ASY male Bullock's orioles were captured in mistnets with the use of conspecific playback and decoys or by positioning nets near oriole feeders. A single tail feather (R3) was collected from each individual during the summers (May through July) of 2012 (*n* = 18) and 2013 (*n* = 9). Identification of the sex and age class was made following descriptions provided by Rohwer and Manning (1990) and Pyle (1997). Each bird was banded with a single Canadian Wildlife Service‐issued aluminum band and a unique combination of three color bands for individual identification.

### 2.3 Stable isotope analysis

To examine diet and habitat during molt, we looked at stable isotopes of both carbon (δ^13^C) and nitrogen (δ^15^N). Carbon (δ^13^C) stable isotope values can indicate habitat type due to differences in plant water stress and photosynthetic systems (Lajtha & Marshall, 1994). Specifically, low δ^13^C values indicate a diet containing high amounts of C_3_ plants (or prey that feeds on C_3_ plants) and plants experiencing little water stress, while high δ^13^C values indicate diets with more C_4_ plants and/or greater water stress (Lajtha & Marshall, 1994). Differing δ^13^C values can also be indicative of marine versus terrestrial dietary input (Lajtha & Marshall, 1994).

Nitrogen (δ^15^N) stable isotope values are often used in food web studies, as they are associated with the trophic level of an organism. ^15^N is preferentially incorporated with increasing trophic level, resulting in greater δ^15^N values at higher trophic levels (Post, 2002; Poupin et al., 2011). Furthermore, δ^15^N values are negatively correlated with rainfall and positively associated with temperature, so that δ^15^N level may be indicative of the temperature and aridity of a biome (Craine et al., 2009; Sealy, van der Merwe, Lee‐Thorp, & Lanham, 1987).

For all feathers, analysis of stable‐carbon and stable‐nitrogen isotope ratios was performed at the Smithsonian Institution Isotope Mass Spectrometry Lab in Suitland, Maryland, USA. Feathers and claws were washed in a 2:1 chloroform‐methanol solution and allowed to dry. Approximately 0.30–0.40 mg of the distal tip of the feather was sampled, excluding the rachis. Using a Thermo high‐temperature conversion elemental analyzer (TC/EA; Thermo Scientific, Waltham, Massachusetts, USA) at 1350°C, samples were first pyrolyzed and sequentially analyzed by a Thermo Delta V Advantage isotope ratio mass spectrometer. We ran two in‐house standards (acetanilide and urea) for every 10 samples. All isotope ratios are reported in δ notation in units per mil (‰) relative to international standards PDB (carbon) and air (nitrogen). Based on repeated measurements of standards, repeatability of carbon and nitrogen samples was ±0.2‰.

### 2.4 Color analysis

Following Reudink, Marra, Boag, and Ratcliffe (2009), reflectance spectrometry was performed on feathers using an Ocean Optics JAZ spectrometer (FL, USA) attached to a PX‐2 xenon pulsed light source to record reflectance spectra across the bird visual spectrum (300–700 nm) from which the variables of hue, red chroma, and brightness were calculated using the R‐based color analysis program RCLR v2.8 (Montgomerie, 2008). Feathers were mounted on a minimally reflective black background (<5% reflectance), and the probe was kept at a 90° angle. Ten measurements were taken haphazardly (i.e., semirandomly) across the widest region of the tail feather, avoiding the rachis and feather edges. In between measurements for each bird, readings from dark (sealed, black velvet lined box) and white (Spectralon; Labsphere, NH, USA) standards were used for standardization. Hue, which describes the color of the feather (i.e., yellow, orange, red), was calculated as arctan ([(*R*_510‐605_ – *R*_320‐415_)/*R*_320‐700_]/[(*R*_605‐700_ – *R*_415‐510_)/*R*_320‐700_]). Red chroma, which describes the degree of color saturation, was calculated as the amount of red light reflected relative to the overall reflectance: (*R*_575‐700_)/(*R*_300‐700_). Brightness was calculated as the mean amount of light reflected across all wavelengths: (mean *R*_300‐700_).

### 2.5 Carotenoid analysis

Carotenoid standards (Sigma‐Aldrich, Oakville, ON, Canada) were made by weighing out canthaxanthin, lutein, or zeaxanthin into a volumetric flask and filling with a chloroform (CHCl_3_) and methanol (MeOH) solution (1:1, v/v). Standards were serial diluted, ranging from 3 to 100 ppm.

Feathers were blotched with hexane and allowed to dry. Once dry, feathers were cut and weighed into glass vials. Antibumping granules were added along with acidic pyridine (three drops of HCl in 50 ml pyridine). The mixture was refluxed for 3 hr. Once cooled, the carotenoids were transferred to hexane by adding hexane and mixing, then washing three times with water. The hexane layer was transferred to a new vial and the drying agent sodium sulfate was added. The extraction was evaporated to dryness with nitrogen gas, and then a known amount of CHCl_3_ and MeOH solution (1:1, v/v) was added. The antioxidant butylated hydroxytoluene (BHT) was also added to prevent oxidation of the carotenoids (2 μl of 10 mg/ml BHT per sample). The extractions were kept in glass vials and stored in a dark freezer until use.

Analysis was performed on an Agilent 1200 series HPLC system (Agilent Technologies, Mississauga, ON, Canada) coupled to an Agilent 6530 Accurate‐Mass Quadrupole Time‐of‐Flight (Q‐TOF) spectrometer, equipped with electrospray ionization (ESI) source (gas temperature, 300°C; drying gas, 8 L/min; nebulizer 35 psig; sheath gas temperature, 350°C; sheath gas flow, 11 L/min; Vcap, 3500 V). Carotenoids were analyzed in positive ion mode, and mass spectra were collected between 100 and 600 m/z. Samples of 5 μl of blanks, extractions, and standards (3–100 ppm) were injected into the LC, with the flow rate set at 1.0 ml/min. Separation was achieved with a Luna C8 (2) column (100 mm × 4.6 mm; 3 μm particle size; Agilent, Canada) kept at a constant temperature of 60 ± 0.2°C. The autosampler and column were set for 60°C. Mobile phase (A) consisted of pure methanol and mobile phase (B) was composed of 70:30 v/v methanol with 0.1% ammonium acetate. Gradient elution was programed as follows: 95% B at time zero, 80% B at 10 min, 65% B at 15 min, 40% B at 20 min, 10% B at 24 min, and 95% B again at 25 min, with the effluent flowing into the Q‐TOF MS.

Data were obtained by taking the area divided by the retention time of the peak of interest. Using the data produced from the standards, a calibration curve was produced for each of canthaxanthin, lutein, and zeaxanthin. Calibration curves were used to determine the given carotenoid content in the extraction sample, which in turn was used to determine the amount of carotenoid in μg per mg of feather extracted. We obtained carotenoid data for *n* = 21 of the 27 Bullock's orioles captured in the field.

### 2.6 Statistical analysis

We used Pearson correlation to examine relationships between brightness, red chroma, and hue and (1) carotenoid concentration and (2) the proportion of each specific carotenoid relative to the total concentration of all three carotenoids. To examine whether carbon or nitrogen stable isotope values predicted either carotenoid content (concentration) or the proportion of each carotenoid, we constructed linear mixed models with carotenoid concentration or the proportion of each carotenoid as the response variable, δ^13^C, δ^15^N, and year as fixed effects, and individual as a random effect (due to repeated measurements of three individuals in different years). We created a similar linear mixed model to examine whether carbon or nitrogen stable isotope values predicted feather color (hue, red chroma, brightness), using feather color as the response variable, δ^13^C, δ^15^N, and year as fixed effects, and individual as a random effect. All statistical analyses were performed using JMP v12.0 (SAS 2012).

## 3 Results

### 3.1 Carotenoid concentration and composition

Average Total feather carotenoid concentration was 2.06 ± 0.86 μg/mg *SD* for the 21 ASY Bullock's orioles from which we were able to obtain carotenoid data. Lutein was the most abundant carotenoid measured in Bullock's oriole feathers (0.99 ± 0.66 μg/mg *SD*), followed by zeaxanthin (0.76 ± 0.23 μg/mg *SD*) and canthaxanthin (0.31 ± 0.10 μg/mg *SD*).

### 3.2 Feather color, carotenoid concentration, and composition

We examined the relationship of (1) carotenoid content and (2) proportion of each carotenoid to the plumage color measurements of hue, red chroma, and brightness (Table 1). We observed no relationship between any of the color variables and the concentration of any carotenoid; however, the proportion of canthaxanthin was associated with increased brightness and more orange‐shifted hue, while a higher proportion of lutein was associated with lower brightness and more yellow‐shifted hue. Zeaxanthin was positively associated with brightness (Table 1).

**Table 1:** Relationships between tail coloration and carotenoid concentration and composition (*n* = 21)

	Brightness	Red chroma	Hue
Total carotenoid	*r* = −.01 *p* = .67	*r* = .12 *p* = .61	*r* = .17 *p* = .45
Canthaxanthin	*r* = .43 *p* = .05	*r* = −.16 *p* = .49	*r* = −.28 *p* = .21
Lutein	*r* = −.29 *p* = .20	*r* = .20 *p* = .38	*r* = .32 *p* = .15
Zeaxanthin	*r* = 28 *p* = .22	*r* = −.06 *p* = .80	*r* = −.15 *p* = .51
% Canthaxanthin	*r* = .60 ***p*** ** = .004**	*r* = −.37 *p* = .10	*r* = −.46 ***p*** ** = .04**
% Lutein	*r* = −.57 ***p*** ** = .007**	*r* = .32 *p* = .16	*r* = .43 ***p*** ** = .05**
% Zeaxanthin	*r* = .55 ***p*** ** = .01**	*r* = −.64 *p* = .21	*r* = −.42 *p* = .06

Bold values indicate significance at α = 0.05

### 3.3 Feather isotopes (δ^13^C, δ^15^N) and carotenoid concentration, composition, and plumage color

**Figure 1:**
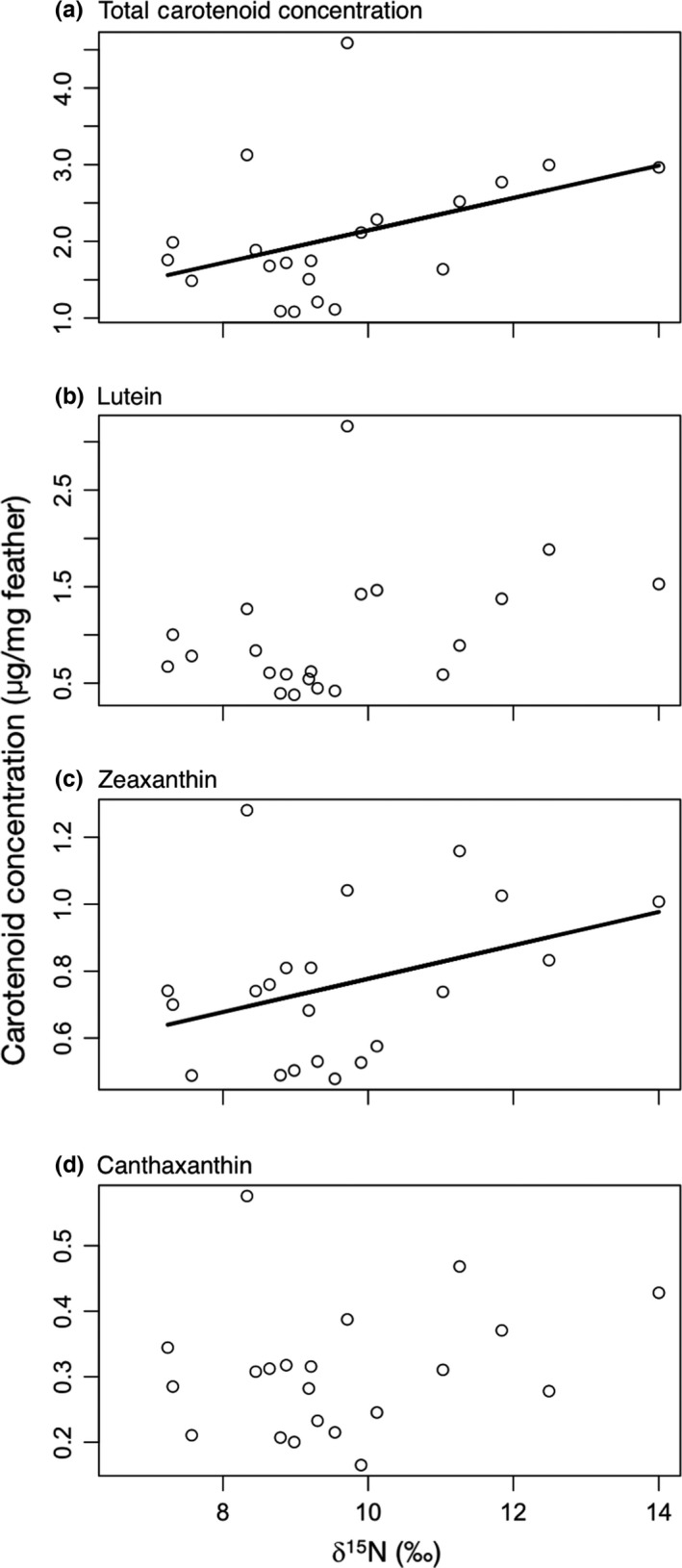
Tail feather δ^15^N values were positively associated with (a) total carotenoid concentration and (c) zeaxanthin concentration and were marginally, but not significantly, associated with (b) lutein and (d) canthaxanthin concentration

**Figure 2:**
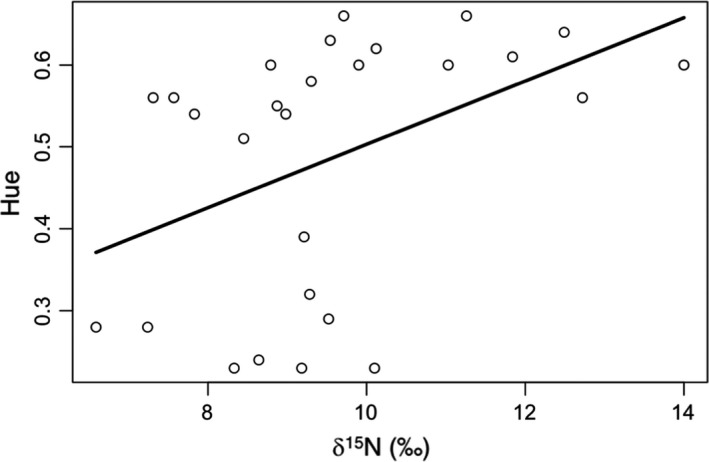
Lower δ^15^N values in tail feathers were associated with more orange‐shifted hue

We asked whether carotenoid concentration or the proportion of canthaxanthin, lutein, and zeaxanthin in the feathers was predicted by feather isotope values. δ^13^C was not associated with any measure of carotenoid concentration or composition; however, δ^15^N was significantly associated with total carotenoid composition and zeaxanthin concentration and marginally, but not significantly, associated with canthaxanthin and lutein concentrations (Table 2; Figure 1). There was also an effect of year for total carotenoid, canthaxanthin, and zeaxanthin concentration. Next, we examined whether δ^13^C and δ^15^N were predictors of feather coloration. Neither brightness nor red chroma was associated with feather isotope values; however, feathers with low δ^15^N values had more orange‐shifted hue (Table 2; Figure 2).

**Table 2:** Relationships between feather isotopes (δ^13^C, δ^15^N) and carotenoid concentration and composition (top) and feather color (bottom)

	δ^13^C	δ^15^N	Year
Carotenoid			
Total carotenoid	Est: 0.03 ± 0.09	Est: 0.24 ± 0.11	Est: −0.22 ± 0.09
	*F*_1,17_ = 0.09	*F*_1,17_ = 4.60	*F*_1,8.77_ = 6.19
	*p* = .77	***p*** ** = .047**	***p*** ** = .035**
Canthaxanthin	Est: −0.004 ± 0.01	Est: 0.02 ± 0.01	Est: −0.04 ± 0.01
	*F*_1,17_ = 0.18	*F*_1,17_ = 3.03	*F*_1,5.29_ = 17.47
	*p* = .67	*p* = .10	***p*** ** = .008**
Lutein	Est: 0.45 ± 1.89	Est: 0.15 ± 0.07	Est: −0.05 ± 0.07
	*F*_1,17_ = 0.28	*F*_1,17_ = 2.73	*F*_1,6.91_ = 0.06
	*p* = .61	*p* = .12	*p* = .46
Zeaxanthin	Est: −0.007 ± 0.59	Est: 0.07 ± 0.03	Est: −0.12 ± 0.02
	*F*_1,17_ = 0.09	*F*_1,17_ = 6.53	*F*_1,13.86_ = 31.89
	*p* = .76	***p*** ** = .02**	***p*** ** < .0001**
% Canthaxanthin	Est: −0.002 ± 0.12	Est: −0.005 ± 0.006	Est: −0.01 ± 0.02
	*F*_1,16.97_ = 0.25	*F*_1,16.87_ = 0.86	*F*_1,4.37_ = 0.42
	*p* = .62	*p* = .37	*p* = .55
% Lutein	Est: 0.01 ± 0.34	Est: 0.01 ± 0.02	Est: 0.02 ± 0.02
	*F*_1,16.94_ = 0.77	*F*_1,16.99_ = 0.77	*F*_1,0.33_ = 0.66
	*p* = .39	*p* = .39	*p* = .71
% Zeaxanthin	Est: −0.007 ± 0.23	Est: 0.007 ± 0.01	Est: −0.02 ± 0.008
	*F*_1,17_ = 0.73	*F*_1,17_ = 0.45	*F*_1,8.46_ = 4.15
	*p* = .41	*p* = .51	*p* = .07
Color			
Brightness	Est: −0.0005 ± 0.05	Est: −0.001 ± 0.002	Est: −0.02 ± 0.004
	*F*_1,21.33_ = 0.07	*F*_1,22.91_ = 0.44	*F*_1,21.61_ = 41.18
	*p* = .80	*p* = .51	***p*** ** < .0001**
Red chroma	Est: −0.004 ± 0.003	Est: −0.004 ± 0.003	Est: 0.01 ± 0.006
	*F*_1,22.71_ = 2.31	*F*_1,22.98_ = 1.24	*F*_1,17.92_ = 3.92
	*p* = .14	*p* = .28	*p* = .06
Hue	Est: 0.0006 ± 0.13	Est: 0.01 ± 0.006	Est: 0.14 ± 0.01
	*F*_1,19.62_ = 0.01	*F*_1,22.55_ = 5.55	*F*_1,21.61_ = 176.31
	*p* = .91	***p*** = **.03**	***p*** ** < .0001**

Bold values indicate significance at α = 0.05

## 4 Discussion

To our knowledge, this study is the first to demonstrate that conditions experienced during molt stopover, occurring hundreds to thousands of kilometers from the breeding grounds, correlate with the acquisition of an important ornamental signal in a songbird. Because of the importance of obtaining colorful plumage for both intra‐ and intersexual communication, individuals should seek the highest quality, most nutrient‐rich environment in which to molt in order to achieve optimal plumage coloration. Thus, while the molt‐migration strategy may have evolved to compensate for low postbreeding food availability in the arid West (Rohwer et al., 2005), habitat selection by individuals within the general stopover region may also be critical for obtaining high‐quality plumage, which may then carryover to influence individual success in subsequent seasons.

Environmental conditions on the molting grounds, as inferred by stable isotope analysis, correlated with both feather color and carotenoid content; however, the nature of the links between environmental conditions, carotenoid intake, carotenoid metabolism, and color expression will require further investigation. Individuals with higher total carotenoid and zeaxanthin concentrations had high δ^15^N values, which are associated with eating at higher trophic levels, low rainfall, and high temperatures, suggesting that one or more of these factors may be associated with carotenoid acquisition. However, lower δ^15^N values (associated with lower trophic levels, higher rainfall, and lower temperatures) were associated with more orange‐shifted hue. This seemingly contradictory finding may indicate that dietary carotenoids were not limited on the molting grounds in any habitat, but that individuals with diets (e.g., high fruit intake relative to insects) or residing in habitats that resulted in lower δ^15^N values were better able to metabolically convert dietary carotenoids into red keto‐carotenoids (McGraw, 2006), perhaps because they were in better condition. Future work is clearly needed to disentangle these complex patterns.

We also detected significant year effects; the oriole feathers collected in 2013 (which had been molted in 2012) had lower δ^15^N values, higher deposits of carotenoids and expressed more orange‐shifted hue than those collected in 2012 (molted in 2011). One possibility is that birds sampled in 2013 were more reliant on fruit in their diet or molted in cooler, wetter environments. However, September 2011 experienced substantially higher rainfall during the monsoon season than 2012 (14.2 cm compared to 0.97 cm; Tuscon, AZ, USA, http://www.wrh.noaa.gov/twc/monsoon/monsoon.php), and thus, one might predict that the high rainfall year (2011) should have resulted in the opposite pattern. Critically though, the Mexican monsoon region encompasses a vast area and molt‐migrant birds appear to have low molt site fidelity. For example, Chambers et al. (2011) found that molt‐migrants in southeastern Arizona were more likely to be detected in riparian areas in a dry monsoon season, suggesting these birds track habitat and resource availability. Regardless, across years, low δ^15^N values were associated with more orange‐shifted hue, but lower carotenoid content. Based on the extensive literature on American redstarts, overwintering in cooler, wetter habitats is associated with better condition and lower stress level (Marra, Hobson, & Holmes, 1998; Angelier et al. 2009), as well as higher interannual survival (Studds and Marra 2005). One possibility is that carotenoids may not be limited during molt, but rather, orioles molting in cooler, wetter habitats might be in better condition, enabling them to make costly metabolic conversions and produce more colorful plumage (Hill, 2011, 2014; Hill & Johnson, 2012; Johnson & Hill, 2013).

Carryover effects (events occurring during one season that affect performance in a following season) can drive differences in fitness through their impact on survival and reproduction (Harrison, Blount, Inger, Norris, & Bearhop, 2011). For example, in the American redstart, acquisition of a high‐quality winter territory results in earlier departure from the tropical wintering grounds (Reudink, Marra, Kyser, et al., 2009), earlier arrival on the breeding grounds (Marra et al., 1998; Norris, Marra, Kyser, Sherry, & Ratcliffe, 2004; Reudink, Marra, Kyser, et al., 2009), and consequently, positively influences reproductive success (Norris et al., 2004; Reudink, Marra, Kyser, et al., 2009). While the literature on carryover effects has grown rapidly in recent years (Harrison et al., 2011), very few studies have examined the potential carryover effects of conditions experienced during molt to subsequent seasons, despite the fact that any such effects may substantially influence the process of sexual selection. Saino, Szep, Ambrosini, Romano, and Møller (2004) showed that trans‐Saharan migratory barn swallows (*Hirundo rustica*), which undergo a complete molt on their wintering grounds in sub‐Saharan Africa, returned to the breeding grounds with shorter tail ornaments following years with high normalized difference vegetation index (NDVI) values, an index indicative of low primary productivity. This finding is particularly important because tail ornament length is directly linked to reproduction (Saino et al., 2004). Similarly, ornament production has been linked to over‐wintering conditions in both pied (*Ficedula hypoleuca*) and collared flycatchers (*Ficedula albicollis*), which also molt in sub‐Saharan Africa (Garant, Sheldon, & Gustafsson, 2004; Järvistö, Calhim, Schuett, & Laaksonen, 2016). In North America, yellow warblers (*Setophaga petechia*) undergo a pre‐alternate molt of body feathers on the wintering grounds; birds overwintering in higher‐quality habitat (inferred via stable isotope analysis) produced more colorful feathers (higher chroma), which are important for mate choice during breeding (Jones, Drake, & Green, 2014). Though American redstarts molt near the breeding grounds, Reudink et al. (2015) demonstrated that over an 11‐year period, the amount of rainfall experienced during the postbreeding molt was associated with carotenoid‐based tail coloration in the subsequent season. In this species, coloration appears to have important signaling functions during both the breeding (Reudink, Marra, Boag, et al. (2009)) and nonbreeding seasons (Reudink, Studds, Kyser, Marra, & Ratcliffe, 2009; but see Tonra et al., 2014). The limited number of studies examining the potential for carryover effects to influence sexual selection by affecting ornament production is perhaps surprising given the immense body of literature on the function and evolution of plumage coloration. Our study thus represents an important line of evidence demonstrating that conditions experienced during stopover molt may have important carryover effects, which have the potential to influence mate choice and the process of sexual selection.

A large body of literature has clearly demonstrated the importance of carotenoid‐based plumage coloration for inter and intrasexual communication. Here, we demonstrate that in Bullock's orioles, plumage coloration, which is directly linked to carotenoid content, is associated with conditions experienced during molt in the Mexican monsoon region. Stopover molt in the Mexican monsoon appears to have evolved independently several times, likely because of harsh late summer conditions and low food availability in western North America; our results suggest that individuals may be able to optimize plumage coloration through the selection of high‐quality habitat within this region. Habitat selection and conditions during molt may therefore represent an important, but largely unstudied, carryover effect influencing sexual selection in the subsequent breeding season.

## Conflict of Interest

None declared.

## Data Accessibility

Once published, data for this paper will be available from DRYAD (datadryad.org).
